# Exploring the experience of patients who receive a telephone follow-up call from intensive care unit nurse specialists following hospital discharge: A descriptive survey

**DOI:** 10.1016/j.ijnsa.2025.100429

**Published:** 2025-09-24

**Authors:** Sian Ingham, Alasdair Jubb, Monica Trivedi, Milena Georgieva, Catherine Yates, Jill Hyde, Joy McAdam, Robyn Davies, Petra Polgarova, Lisa Enoch, Olivia Bentham, Eleanor Ruffle, Joanne McPeake

**Affiliations:** a Cambridge University Hospitals NHS Foundation Trust, Cambridge, UK; b The Healthcare Improvement Studies Institute, University of Cambridge, Cambridge, UK

**Keywords:** Critical illness, Recovery and rehabilitation, Nurse led follow-up and family

## Abstract

**Background:**

Following hospital discharge from a critical care-related admission, patients can experience multiple problems. There is limited data about how patients can effectively be supported during recovery from critical illness. As such, different services have emerged internationally with the aim of improving outcomes.

**Objective:**

The objective was to understand the experience of patients who received a telephone follow-up call from an Intensive Care Unit Recovery Nurse Specialist following a critical care-related hospitalisation. We sought to understand what patients valued about these calls, alongside any improvements which could be made.

**Methods:**

Patients from a single centre in the UK, who received a telephone call by the Intensive Care Unit Recovery Nurse Specialist team approximately one month following hospital discharge, were asked to provide feedback on the call via a pre-specified survey. This study employed a descriptive design to report findings from the survey, integrating quantitative and qualitative data to comprehensively explore participant experiences. In this analysis, qualitative feedback was analysed using Framework Analysis and descriptive statistics were used to analyse quantitative data.

**Results:**

We analysed survey data from 125 patients; this represented a response rate of 28.5%. In total, 98.2% (*n* = 123) of respondents found the call very useful or somewhat useful and 97.1% (*n* = 121) of respondents stated that the telephone call addressed or somewhat addressed physical health needs and 96.2 % (*n* = 120) of respondents stated that the telephone call addressed or somewhat addressed emotional health needs. From the qualitative data we derived five themes related to the experience of those receiving these telephone follow-up calls: reassurance and validation; personalised care planning and signposting; access to expertise; family support and scheduling and planning. Potential improvements included: the ability to schedule the call and the purposeful involvement of family members in the process.

**Conclusions:**

This single centre analysis has explored the experience of those receiving a telephone follow-up call from an Intensive Care Unit Recovery Nurse Specialist following critical illness. In the respondents of this evaluation, the call provided reassurance, alongside supporting navigation to appropriate services following hospital discharge. Future research should explore the effectiveness of this intervention, including its impact across diverse groups.

**What is already known**

• Patients can experience multiple problems following critical illness
• There is limited data on how patients can best be supported to improve functional outcomes following critical illness
• Telephone follow up can take multiple formats during recovery from intensive care; however, there is limited data on the efficacy or effectiveness of telephone follow-up. There is also limited experience data from patients involved in these services.

Alt-text: Unlabelled box

**What this paper adds**

• Respondents in this single-centre evaluation highlighted that they felt reassured following a call, moreover, they highlighted that this access to expertise was highly valued.
• This evaluation also highlighted the importance of supporting family members during recovery from critical illness to ensure optimal recovery for the patient.
• Future research should explore whether this intervention can have a meaningful impact on patient and family member outcomes.

Alt-text: Unlabelled box

## 1 Introduction

It is well established that survivors of critical illness can experience multiple problems (McPeake et al., 2020). These problems are complex and can be physical, social, emotional and cognitive in nature (Hauschildt et al., 2020; Marra et al., 2018). These ongoing problems, which are thought to impact up to 80 % survivors are wide-ranging (Marra et al., 2018). Psychological problems include anxiety, depression and symptoms of Post-Traumatic Stress (PTSD) (McPeake et al., 2021). Physical challenges include chronic pain and mobility issues, while cognitive impairments in memory and executive functioning may affect almost half of survivors (Devine et al., 2019; Iwashyna et al., 2010). As well as having a profound impact on the individual and their families, survivorship can also have a substantial impact on the healthcare system and society (Mikkelsen et al., 2020). Recent data has shown that up to 30 % of critical care survivors are readmitted to hospital within 90 days of hospital discharge and approximately half do not return to employment six months after critical illness (McPeake et al., 2019, 2022). Survivors of critical illness are also more likely to require government funded welfare support following hospital discharge, compared to non- critical care hospital control comparators (J McPeake et al., 2021).

Despite the vast evidence base describing the challenges which survivors of critical illness face, there is limited data on how people can best be supported to improve functional outcomes (Schofield-Robinson et al., 2018). As a result, multiple different services and approaches to care planning have emerged internationally, leading to significant care variation and potential inequalities in care access (Connolly et al., 2021). These services include recovery clinics and peer support programmes (J McPeake et al., 2021). Other approaches have been implemented but have received minimal evaluation in the literature. One such approach is telephone follow-up.

Telephone follow-up is delivered in multiple formats during recovery from intensive care; this can include multi-disciplinary calls or a call from a single discipline such as nursing (Teixeira and Rosa, 2018). Calls can vary in nature but tend to provide an assessment for new or worsening impairments and signpost survivors to other Intensive Care Unit recovery or community services. However, there is limited data on the efficacy of these services. There is also limited experience data from patients who receive these calls following hospital discharge (Nakanishi et al., 2024).

To understand the experience of patients receiving nurse-led telephone follow-up following critical care discharge, we undertook a service evaluation of routinely collected survey data. We also explored what people valued about these calls, alongside any improvements which could be made.

## 2 Methods

### 2.1 Approvals

Approval for this analysis was obtained from Cambridge University Hospital's Patient Outcomes team. We explored routinely collected, anonymised patient experience data. Permission was obtained on 10/07/24 (registration number 12,325).

### 2.2 Design

We sought to understand the experience of patients who received nurse-led telephone follow-up (the intervention) following critical care discharge in a single centre in the UK. We evaluated the experience of these patients by distributing a digital survey following the telephone call. The survey included open and closed questions (Table 1). This service evaluation employed a descriptive design to report findings integrating quantitative and qualitative data to comprehensively explore participant experiences and outcomes.

**Table 1:** Survey questions and potential responses.

**1.** Date of your appointment.
**2.** Were you expecting the telephone call? (Yes/No)
**3.** Would you have preferred an appointment date/time? (Yes/No)
**4.** Please share your thoughts on having a date for your telephone appointment (Open response)
**5.** Do you feel the telephone appointment addressed your physical health? (Yes/Somewhat/No)
**6.** Do you feel the telephone appointment addressed your emotional health? (Yes/Somewhat/No)
**7.** Please share your thoughts on the telephone call addressing your physical and emotional health (Open response)
**8.** Did you have any symptoms you were concerned about before the telephone appointment? (Not Sure/No/Yes)
**9.** Do you feel the telephone call addressed these symptoms? (Yes/Somewhat/No)
**10.** Are there questions you feel we should have asked during the telephone assessment? (Not Sure/No/Yes)
**11.** Please share your thoughts on the questions we asked, or questions you feel we should have asked (Open response)
**12.** Was the telephone call the right length of time? (Too short/too long/just right)
**13.** Did you find the telephone call useful? (Not useful at all/somewhat useful/very useful)
**14.** Please tell us what you feel we did well during the telephone appointment (Open response)
**15.** Please tell us what you feel we did not do well during the telephone appointment (Open response)
**16.** Please share any additional thoughts on how we could improve the critical care telephone appointment for future patients (Open response)
**17.** Gender.
**18.** Age.
**19.** How would you describe your ethnic group?
**20.** Do you have a disability?
**21.** If yes, please tick as many boxes as apply.

### 2.3 Population

Patients admitted to two Intensive Care Units were included; one general surgical/medical and one neurosurgical and trauma Intensive Care Unit. Patients were eligible for telephone follow-up if they had a critical care stay of 10 days or more. Patients who were deemed to be at high risk of problems following hospital discharge, highlighted via a ward visit by the Intensive Care Unit Recovery Nurse Specialist team were also contacted via telephone.

### 2.4 Intervention

Intensive Care Unit Recovery Nurse Specialists, with experience of critical care recovery, called patients approximately one month following hospital discharge. Two call attempts were made, if the patient could not be reached, a letter was sent detailing the contact information of the Intensive Care Unit Recovery team. Patients who were receiving ongoing rehabilitation as an inpatient could have a call postponed until they are able to be fully assessed.

During the calls, which took approximately 45 min, a recovery assessment, underpinned by the Post- Intensive Care Unit Presentation Screen (PICUPS)- community tool was made (Turner-Stokes et al., 2022). This included a discussion of emotional, physical, and cognitive recovery problems. If families were present during the calls, they were encouraged to participate (with the direct consent of patients). If the patient’s first language is not English, a family member supports the call or a hospital translator is organised.

Depending on the discussion, patients with ongoing issues were offered an appointment at the Intensive Care Unit follow-up clinic within the hospital, or a visit to the Intensive Care Unit. The team also provided signposting to local community organisations (for example to brain injury charities or charities which support informal carers). If a patient did not require all services provided by the Intensive Care Unit follow-up clinic, specialist referrals could be made to individual services such as dietetics for nutritional support.

### 2.5 Data collection

During the telephone call the Intensive Care Unit Specialist Nurse checked that the patient was happy to receive a routine follow-up survey via email (digital survey) to explore their experiences of the follow-up call. A hospital administrator then emailed patients a link to a standardised patient experience survey within 48 h of the call, if the patient was happy to receive the survey. The content of this survey was designed by the hospital patient experience and the Intensive Care Unit recovery team. This survey includes a range of demographic, closed and open questions. A copy of the survey questions included in this analysis is provided in Table 1.

Demographic data for all people who received telephone follow-up calls during the study period was collected. Demographic data included: sex, ethnicity, and age.

### 2.6 Data analysis

Quantitative questions were analysed using summary statistics. Framework analysis was employed to analyse the qualitative, open ended questions, due to the succinct format of the data (Gale et al., 2013). No theoretical framework was used to underpin the Framework analysis. All data was anonymized before analysis by the hospital patient experience team.

There are seven stages to framework analysis: (1) transcription; (2) familiarization with the interview; (3) coding; (4) developing a working analytical framework; (5) applying the analytical framework; (6) charting data into the framework matrix; (7) interpreting the data (Gale et al., 2013).

Two researchers (JM and SI), both experienced critical care nurses, undertook preliminary sweeps of the data to familiarize themselves with the content and develop initial coding. The data was grouped manually. The two researchers then jointly developed a working analytical framework. The analytical framework was rechecked against the preliminary analyses and raw data; a framework matrix was then created. A clear audit trail of decision making, and the coding framework development was kept and reviewed at each stage. To ensure rigor and trustworthiness, crosschecking of the analyses and audit trail was undertaken independently by another member of the research team (CY).

## 3 Results

Survey data was collected between 1st of May 2022 until 31st of March 2024. 438 people received phone calls from the Intensive Care Unit Recovery Nurse Specialist team during this period. Of this cohort, 262 (60 %) respondents were male, and the most common age bracket represented was 53 - 67 years (*n* = 149, 34 %). The majority of the respondents were White/British (*n* = 342, 78 %). Full demographic details are provided in Table 2. Data from 125 survey respondents (representing a 28.5 % response rate) were included in this analysis.

**Table 2:** Participant demographics. Demographics of people receiving telephone follow up by nurse specialists.

Demographic	*N* = 438 ( %)
**Sex:**	
Male	262 (60)
Female	176 (40)
**Age:**	
17–37 years	81 (18.5)
38–52 years	102 (23.3)
53–67 years	149 (34.0)
68 years and older	106 (24.2)
**Ethnicity:**	
White British or Irish	344 (78.5)
Asian (Bangladeshi, Indian, Pakistani)	9 (2.1)
Black (African and Caribbean)	6 (1.4)
Mixed White (Black African and Black Caribbean)	5 (1.1)
Other Background	42 (9.6)
Not stated	32 (7.3)

In terms of quantitative feedback from the call, 98.2 % (*n* = 123) of respondents found the call very useful or somewhat useful and 96.0 % (*n* = 120) felt that the telephone call was the ‘right length of time’. Across the survey 97.1 % (*n* = 121) of respondents stated that the telephone call addressed or somewhat addressed physical health needs and 96.2 % (*n* = 120) of respondents stated that the telephone call addressed or somewhat addressed emotional health needs.

We derived five themes related to the experience of those receiving telephone follow-up calls from the qualitative, open-ended questions: reassurance and validation; personalised care planning and signposting; access to expertise; family support and scheduling and planning. Potential improvements included: the ability to schedule the call and the purposeful involvement of family members in the process. A selection of representative quotes from across survey respondents are presented in Table 3.

**Table 3:** Table of quotes illustrating the themes generated from this analysis. Illustrative quotes extracted from survey responses.

Theme	Feedback quotes
**Reassurance and Validation**	“Very good at listening to emotional and physical health problems and rationalising them for me.” “Good to know my situation was common amongst others who had same circumstances.” “The phone call helped me with a lot of questions. The answers to these questions made me feel better about the way I was feeling.” “I felt the call was a good way of looking at what had happened with me over the time I was in hospital and that it helped me to see where I was today. I found the offer of going to the hospital to learn more about what happened to me in ICU to be a positive way of understanding what had happened.”
**Family support**	“The subject range and topics covered were all very relevant to my situation and very useful. I do not believe there are additional questions that I have but it would be useful to include my wife in such discussions as she was present a lot more than I was and she has many questions.” “I think it was a very comprehensive telephone call. I felt that there was time for me to talk and raise any concerns, which was helpful. I think it may be pertinent to ask about my relationship with my wife since she has been on a very different journey to me and we are still working all of that out, despite being married for over 40 years. There is a lot of emphasis on the patient but part of recovery depends on the support of loved ones who have also been through a lot and are dealing with the own emotional recovery.” “The caller was helpful but I was discharged in December and it was difficult to recall my time in ICU and on the wards. She was going to chase up the pain team about my follow up appointment. She told me about help my family could have if they were badly affected by seeing me in ICU. In all she was very helpful. She also followed up asking the dietician to phone me about trying to gain the weight I had lost. They rang yesterday which was excellent.”
**Personalised care planning and signposting**	“Very helpful, shows a real interest in my health issues.” “I have no experience of this and so cannot made a comparison but the nurse I spoke with took time to understand me and the root cause of my issues. I’m not a person that opens up easily or freely but I did do so during this call and I have felt 100 % more in control since the call. A thousand thanks.”
**Access to expertise**	“Clear and concise questions that allowed for in depth answers and the utmost care and understanding was there from the beginning to the end of the conversation.” “I felt the caller was well briefed on the issues I had been through, and it helped that she was also able to talk to my wife at the same time.” “The nurse was extremely thorough with all her questions and listened to my answers carefully to enable her to give me the best response possible.”
**Scheduling and planning**	“I possibly would have been mentally prepared for the questions and had my own questions ready rather than being put on the spot which doesn’t give you time to think thoroughly.” “I’m recovering at home so would be available, with or without a set date and time.” ‘It needs to be scheduled for when carers can be involved. Many of the patients will have aphasia or lack the confidence to converse on their own.”

### 3.1 Reassurance and validation

Overwhelming survey respondents described the reassurance they felt after receiving the call. For example, one respondent stated:

“I felt reassured and listened to throughout the call. It was a real positive experience but also a vital service to offer and the feeling that the care was extended into rehab too.”

Respondents also described a sense of validation following the call. Specifically, this included understanding the journey that they had been on, as well as contextualising progress. This helped people reflect positively on recovery progress:

“I felt the call was a good way of looking at what had happened with me over the time I was in hospital and that it helped me to see where I was today. I found the offer of going to the hospital to learn more about what happened to me in Intensive Care Unit to be a positive way of understanding what happened.”

### 3.2 Personalised care planning and signposting

An important aspect of this follow-up call was the delivery of recovery care which was specific to the respondent’s recovery journey. As well as pre-specified questions (underpinned by the Post- Intensive Care Unit Presentation Screen (PICUPS)- community tool), survivors have the opportunity to ask questions specific and personal to their recovery journey:

“The caller was very polite, friendly and seemed knowledgeable and was very helpful and supportive. She also advised me on several issues I required guidance on.”

A further component of the calls which appeared to be valued was the signposting to community organisations who could potentially help with recovery. One respondent highlighted this specific support:

“The nurse was very helpful and caring. She gave me information I was unaware of about the self-help groups I could take part in to aid my recovery physically and mentally.”

This service also supported referral to clinical specialists which were deemed necessary for optimal recovery following the telephone assessment. For example, one participant highlighted the impact of the referrals which had been made during the telephone follow-up call:

“Information about help for my family. Information about how recovery can take a year. Offering to chase up the pain team although they have not been in touch. Also offering help from dietician which came yesterday afternoon. The caller made me feel like a person.”

*Access to expertise*

Phone calls were undertaken by experienced Intensive Care Unit Recovery Nurse Specialists who provided people with detailed information about the recovery trajectory in an accessible way. For example:

“The huge benefit to me of engaging with a medical expert/practitioner was the ability to discuss my concerns in an iterative process, where the caller can ask the exact questions which tease out the fundamental issues, and address them in a way that I can easily understand.”

Participants also highlighted the importance of having access to clinicians who had expertise of Intensive Care Unit care as well as recovery and rehabilitation services. For example, one survey participant highlighted the importance of being able to discuss parts of their illness journey which they couldn’t remember:

“The phone conversation was really relevant and specific to how I was recovering, my reflections/memories of the Intensive Care Unit experience and help to address some aspects that were particularly relevant to me regarding the immediate time after surgery for which I was not conscious. I hope the actual meeting will address further some of the questions that my wife in particular has.”

Of note, the majority of survey respondents 96.0 % (*n* = 120) felt that the phone call was the correct length of time (approximately 45 min); this extended access to expertise appeared to be valued:

“I was not rushed. I was allowed to explain things and all my questions were answered as well as being made to feel cared for and that things were progressing nicely.”

*Family support*

Recent evidence has highlighted that family members of survivors can also experience ongoing emotional and social issues following discharge from critical care (Haines et al., 2018). The support given to family members during this call, was valued by respondents of the survey. For example, one respondent stated:

“She told me about help my family could have if they were badly affected by seeing me in ICU. In all, she was very helpful.”

However, the purposeful inclusion and support of family members was also one area which respondents felt could be improved upon. For example, one survey respondent described how the inclusion of family members could have supported the wider social unit:

“I think it was a very comprehensive telephone call. I felt that there was time for me to talk and raise any concerns, which was helpful. I think it may be pertinent to ask about my relationship with my wife since she has been on a very different journey to me and we are still working all of that out, despite being married for over 40 years. There is a lot of emphasis on the patient, but part of recovery depends on the support of loved ones who have also been through a lot and are dealing their own emotional recovery.”

*Scheduling and Planning*

The Intensive Care Unit Recovery Nurse Specialist team did not provide a date or appointment for the call. Before leaving hospital, patients and families would be informed that they would receive the call, but they did not receive a specific time. Although the majority of respondents of this survey (68.7 %, *n* = 86), would not have preferred to have an appointment date or time, the remaining respondents highlighted that having a scheduled call could be an area for improvement to enable them to fully engage and ensure families are present. For example, one respondent stated:

“We did know that we would be contacted at some stage. However, if you have a time/date it saves you the worry of potentially missing the call, especially if you have concerns. It also gives you the opportunity to make a note of anything specific you would like to ask and saves you worrying needlessly about how to take concerns forward. I did not realise that the call/follow would be so wide ranging in content.”

## 4 Discussion

This service evaluation has provided details of the experience of critically ill patients receiving a telephone follow-up call from Intensive Care Unit Recovery Nurse Specialists following hospital discharge. Respondents in this single-centre evaluation highlighted that they felt reassured following a call, moreover, they highlighted that this access to expertise was highly valued. The patients who received these calls also appeared to value personalised care planning and the family support offered. Quantitative data also demonstrated that the majority of patients felt their physical and emotional issues were addressed during the follow-up call. Future research should explore whether this intervention can have a meaningful impact on patient and family member outcomes.

Respondents of this survey appeared to value the telephone call following hospital discharge, however, this intervention is most likely part of a larger jigsaw of recovery. During calls the Intensive Care Unit Recovery Nurse Specialists could offer several other services including ongoing care with the Intensive Care Unit recovery clinic, an Intensive Care Unit visit, referral to other specialist services or signposting to local organisations. Given this, it is likely that some survivors will need ongoing care to enable optimal recovery. This evaluation highlights that a personalised approach to critical care recovery is needed for people discharged from hospital and a single approach or intervention is unlikely to benefit the entire population. At present there is limited evidence to direct clinicians in how to deliver Intensive Care Unit recovery care which improves functional outcomes (Schofield-Robinson et al., 2018; Stewart et al., 2023). Future research should explore how patients could be stratified to receive the care most appropriate to their needs. This could include the use of novel trial methods such as platform trials, which evaluate the effectiveness of multiple interventions, dependent on the individualised need of the patient.

This evaluation also highlighted the importance of supporting family members during recovery from critical illness. Recent evidence has demonstrated that family members can also experience significant emotional and social morbidity in the months following exposure to critical care (Docherty et al., 2024; Sevin et al., 2021). Family members may also need to adopt the role of informal carer, with significant associated challenges (Docherty et al., 2025). While caring for this group is clearly important to their own individual needs, recent research has also highlighted that elevated family and carer strain, measured via validated instruments, is related to hospital readmission in survivors of critical illness (Docherty et al., 2025). Future research should integrate the ongoing needs of family members, to ensure that the patients’ entire social unit is able to thrive in the months following hospitalisation.

Across the survey responses it was clear that respondents valued the expertise of the Intensive Care Unit Recovery Nurse Specialist providing the follow-up. This expertise included the management of symptoms following critical illness (including onward treatment pathways), but also in relation to understanding the Intensive Care Unit experience. In the critical care recovery literature, there has been debate over which specialists should provide care to survivors of critical illness, including whether this should be provided by rehabilitation specialists or community clinicians such as General Practitioners and family physicians rather than Intensive Care Unit clinicians (Meyer et al., 2018). This analysis has demonstrated that patients appear to value having access to expertise of critical illness during recovery. Future interventional research in this area should consider this important concept. Furthermore, future qualitative research should seek to provide a more detailed understanding of the benefits and associated challenges, of accessing recovery services for patients and their family members.

This service evaluation has used a robust approach to data analysis and has included a diverse cohort of patients following critical illness. However, it does have limitations. Firstly, the survey was only available to people who had an email address and access to the internet, with no option for postal input. This approach might have excluded people with limited digital literacy, or those who did not have access to the internet. Secondly, this survey was distributed in a single centre in a single healthcare system (National Health System, UK). As such, it might not be representative of other healthcare systems. Thirdly, as with all surveys, we only have responses from a sub- group of the population; those who did not respond might have different views. Additionally, the survey was distributed in English only, which clearly limits the diversity of people involved in this research. Future work should examine the experience of people who first language is not English. Finally, this survey was designed specifically by this local Intensive Care Unit and patient experience team; it does not represent a validated questionnaire or patient experience tool. Additionally, the patient experience team in this hospital provided anonymised, non-linked quotes for analysis, as such we are not able to link patient data with the quotes provided. Moreover, as this is a patient satisfaction survey, it does not provide robust details of the acceptability and effectiveness of this intervention.

Learning from this service evaluation provides several implications for both clinical practice and future research. Given the limited data on both the effectiveness of recovery services and the experience of people using these services, this evaluation has demonstrated that nurse-led follow up calls appear to be valued by patients, with the majority of participants finding the call useful in their recovery. Future research must now examine the effectiveness of this intervention and its potential impact on outcomes.

In conclusion, this single centre service evaluation has shown that critical care survivors appear to value a telephone follow-up appointment which provides a detailed assessment and further information and care provision. Future research should explore the impact of this intervention on patient outcomes and seek to understand if specific subgroups benefit from this approach to care.

## Funding

This research was supported by the NIHR Cambridge Biomedical Research Centre (NIHR203312). The views expressed are those of the authors and not necessarily those of the NIHR or the Department of Health and Social Care'.**JM is supported by the** Health Foundation's grant to the University of Cambridge for The Healthcare Improvement Studies Institute (THIS Institute). THIS Institute is supported by the Health Foundation, an independent charity committed to bringing about better health and health care for people in the UK.

## CRediT authorship contribution statement

**Sian Ingham:** Writing – review & editing, Writing – original draft, Methodology, Investigation, Formal analysis, Data curation, Conceptualization. **Alasdair Jubb:** Writing – review & editing, Project administration, Investigation, Data curation. **Monica Trivedi:** Writing – review & editing, Data curation. **Milena Georgieva:** Writing – review & editing, Data curation. **Catherine Yates:** Writing – review & editing, Validation, Data curation. **Jill Hyde:** Writing – review & editing, Data curation. **Joy McAdam:** Writing – review & editing, Data curation. **Robyn Davies:** Writing – review & editing, Data curation. **Petra Polgarova:** Writing – review & editing. **Lisa Enoch:** Writing – review & editing, Supervision, Project administration. **Olivia Bentham:** Writing – review & editing, Project administration, Formal analysis, Data curation. **Eleanor Ruffle:** Writing – review & editing, Data curation. **Joanne McPeake:** Writing – review & editing, Writing – original draft, Supervision, Methodology, Formal analysis, Conceptualization.

## Declaration of competing interest

JMcP’s reports that her institution receives consultancy fees from AstraZeneca for work undertaken by JMcP. The other authors declare no potential conflicts of interest.
